# Cognitive and Neuropsychiatric Profiles in Idiopathic Rapid Eye Movement Sleep Behavior Disorder and Parkinson’s Disease

**DOI:** 10.3390/jpm11010051

**Published:** 2021-01-16

**Authors:** Francesca Assogna, Claudio Liguori, Luca Cravello, Lucia Macchiusi, Claudia Belli, Fabio Placidi, Mariangela Pierantozzi, Alessandro Stefani, Bruno Mercuri, Francesca Izzi, Carlo Caltagirone, Nicola B. Mercuri, Francesco E. Pontieri, Gianfranco Spalletta, Clelia Pellicano

**Affiliations:** 1 Fondazione Santa Lucia, IRCCS, 00179 Rome, Italy; f.assogna@hsantalucia.it (F.A.);l.macchiusi@hsantalucia.it (L.M.); c.caltagirone@hsantaluica.it (C.C.); mercurinb@gmail.com (N.B.M.); or francesco.pontieri@uniroma1.it (F.E.P.); g.spalletta@hsantalucia.it (G.S.); 2 Dipartimento di Medicina dei Sistemi, Università “Tor Vergata”, 00133 Rome, Italy; dott.claudioliguori@yahoo.it (C.L.); fbplacidi@gmail.com (F.P.); pierantozzim@gmail.com (M.P.); Stefani@uniroma2.it (A.S.); 3 Centro di Medicina del Sonno, Unità di Neurologia, Università “Tor Vergata”, 00133 Rome, Italy; fraizzi@tin.it; 4 Centro Regionale Alzheimer, ASST Rhodense, 20017, Rho, Italy; lcravello@asst-rhodense.it; 5 Dipartimento di Psicologia, Facoltà di Medicina e Psicologia, “Sapienza” Università di Roma, 00185 Rome, Italy; claudiabelli9595@gmail.com; 6 UOC Neurologia, Azienda Ospedaliera “San Giovanni Addolorata”, 00184 Rome, Italy; hydrargirium65@icloud.com; 7 Dipartimento di Neuroscienze, Salute Mentale e Organi di Senso, “Sapienza” Università di Roma, 00189 Rome, Italy

**Keywords:** neurodegeneration, risk factors, neuropsychiatry, anxiety, depression

## Abstract

Rapid eye movement (REM) sleep behavior disorder (RBD) is a risk factor for developing Parkinson’s disease (PD) and may represent its prodromal state. We compared neuropsychological and neuropsychiatric phenotypes of idiopathic (i) RBD, PD and healthy comparators (HC) in order to identify iRBD specific characteristics. Thirty-eight patients with iRBD, 38 PD patients with RBD (PD + RBD), 38 PD patients without RBD (PD-RBD) and 38 HC underwent a comprehensive neurological, neuropsychological and neuropsychiatric evaluation. iRBD, PD + RBD and PD-RBD performed worse than HC in short-term verbal memory, praxia, language and executive functions. iRBD had higher levels of anxiety, depression, apathy and alexithymia than HC. iRBD had higher levels of apathy than PD + RBD. Both PD groups had higher levels of anxiety and depression than HC. Surprisingly, iRBD performed better than all groups in long-term verbal memory. Patients diagnosed with iRBD are characterized by poor global cognitive performance, but better long-term memory and higher levels of depression, anxiety, alexithymia and apathy. Alexithymia and apathy in patients diagnosed with iRBD may be the expression of precocious derangement of emotional regulation, subsequently observed also in PD. Cognitive and neuropsychiatric symptoms of iRBD are early clinical manifestations of widespread neurodegeneration.

## 1. Introduction

Rapid Eye Movement (REM) sleep Behavior Disorder (RBD) is a parasomnia characterized by loss of normal skeletal muscle atonia during REM sleep, such that patients “act out” dreams, often violently, which is potentially harmful for themselves and their bedpartner [1]. The idiopathic form of RBD (iRBD) generally affects male adults [2]. Up to 80% of patients with iRBD develop a synucleinopathy, namely Parkinson’s disease (PD), dementia with Lewy bodies (LBD) and multiple system atrophy (MSA), with latency from RBD onset to phenoconversion of over 10 years and rates of conversion of 6.25% per year [3]. The prevalence of RBD reaches about 15–60% in patients with PD [4], whereas the prevalence of iRBD is less than 1% in the general population [5]. Several data indicate that RBD has a higher positive likelihood ratio than any other PD prodromal markers, such as olfactory deficits or depressive mood [6]. Namely, RBD can be considered as an early clinical manifestation of future widespread PD neurodegeneration. Indeed, RBD and PD share some pathophysiological mechanisms, such as neuronal loss and α-synuclein degeneration in brainstem nuclei modulating REM sleep atonia, in locus coeruleus-subcoeruleus complex, in the raphe nucleus, substantia nigra and common neuroinflammation markers [7].

Previous studies identified in patients with iRBD [3,8,9,10,11], early signs of neurodegeneration, such as EEG slowing, color vision impairment, olfactory dysfunction, decreased striatal dopamine transporter uptake, substantia nigra hyperechogenicity and reduced cardiac sympathetic innervation. These neurobiological abnormalities are common to those observed in patients with PD; thus, the question is raised of how far cognitive and neuropsychiatric disorders characterizing PD can actually be detected in patients diagnosed with iRBD. Indeed, the latter experienced cognitive dysfunctions affecting different domains, such as visuoperceptive, visuospatial constructional and learning abilities [12,13]; attention, decision making and executive functions [14,15,16]; and working, logical, visual and verbal memory [2,15,17,18,19,20]. Impaired cognitive performance has been considered as a possible marker of prodromal neurodegenerative states [14] in iRBD, but there are not convergent data. Deficits in attention, executive function, decision-making, verbal memory, visuospatial and visuoperceptive abilities were identified also in RBD secondary to PD (PD + RBD) [12,20,21,22]. Conversely, limited studies investigated the neuropsychiatric phenomenology in iRBD and PD + RBD, and showed that both groups experienced definite behaviors disorders, such as apathy, depression and anxiety [23,24,25,26,27].

The aim of the present study is to identify specific neuropsychological and neuropsychiatric features of patients diagnosed with iRBD and to compare their symptoms with those found in HC, PD + RBD and PD without RBD (PD-RBD). Considering iRBD as the prodromal state of synucleinopathy, we anticipated iRBD had a cognitive and neuropsychiatric profile similar to PD + RBD and PD-RBD, different from healthy comparators (HC). In particular, we predicted that patients with iRBD experience executive dysfunctions and motivational and emotional dysregulation.

## 2. Methods

### 2.1. Participants

The study was carried out on 38 patients diagnosed with iRBD, 38 with PD + RBD and 38 with PD-RBD, according to international guidelines [28,29]. All patients were enrolled at the Movement Disorder and Sleep Outpatient Services of our Institutions (Fondazione Santa Lucia IRCCS, Rome, Italy; Department of Neuroscience, Mental Health and Sensory Organs, University “Sapienza,” Sant’Andrea Hospital, Rome, Italy; Sleep Medicine Centre and Department of Medicine of Systems, Neurology Unit, University “Tor Vergata,” Rome, Italy; and Neurology Unit, “San Giovanni Addolorata” Hospital, Rome, Italy) during scheduled visits between January 2015 and December 2018. We also recruited 38 HC in the same geographical area. All participants were one to one pair-matched for gender (100% concordance), age (±1 year) and educational level (±1 year).

Common inclusion criteria for all groups were: (1) age between 55 and 85 years; and (2) vision and hearing sufficient for compliance with testing procedures.

Specific inclusion criteria in iRBD were: (1) iRBD diagnosis made by video-polysomnography (v-PSG) according to International Classification of Sleep Disorders-3rd Edition (ICSD-3) criteria [29]; (2) no sleep-related hypoventilation, pulmonary insufficiency and oxygen desaturation index ≥15/h at v-PSG; (3) absence of diagnosis of PD and/or other neurological disorders, based on examination performed by an expert neurologist; (4) absence of iatrogenic causes of RBD; and (5) no signs of neurodegenerative diseases.

Specific inclusion criteria in patients with PD were: (1) PD diagnosis made in according to the UK Parkinson’s Disease Society Brain Bank diagnostic criteria [28]; (2) Mini-Mental State Examination (MMSE) score ≥26 and no dementia according to the Movement Disorder Society (MDS) clinical diagnostic criteria [30]; and (3) stable dopaminergic therapy for at least 2 months before enrollment.

Common exclusion criteria for all participants enrolled were the following: (1) presence of major medical illnesses (non-stabilized diabetes, obstructive pulmonary disease or asthma, hematologic and oncologic disorders, vitamin B12 or folate deficiency, pernicious anemia, clinically significant and unstable active gastrointestinal, renal, hepatic, endocrine or cardiovascular disorders, and recently treated hypothyroidism); (2) known or suspected history of alcoholism, drug dependence and abuse, head trauma and major psychiatric disorders (apart from mood or anxiety disorders) according to the DSM-V criteria [31]; (3) any potential brain abnormality and microvascular lesion as apparent on conventional fluid attenuated inversion recovery (FLAIR) scans; in particular, the presence, severity, and location of vascular lesions were computed according to the semi-automated method recently published by our group [32]; and (4) concomitant obstructive sleep apnea syndrome based on a validated sleep medicine interview and/or v-PSG (Apnea-Hypopnea Index ≥15/h).

The study was approved by the Ethical Committee of Fondazione Santa Lucia IRCCS and, in accordance with the Helsinki Declaration, each subject signed an informed consent form prior to enrollment.

### 2.2. Sociodemografic And Clinical Assessment

The sociodemographic and neurological features of patients were collected at enrollment by neurologists with expertise on parkinsonism and sleep disorders. The severity of parkinsonian symptoms was measured by the Unified Parkinson’s Disease Rating Scale—part III (UPDRS-III), and PD severity was staged according to the modified Hoehn and Yahr (H&Y) scale [33]. Dopamine replacement therapy was calculated as total daily levodopa equivalents. In the case of dopamine agonists, the following conversion table was used: 1 mg pramipexole = 5 mg ropinirole = 5 mg rotigotine = 100 mg levodopa. The diagnosis of “probable RBD” was performed by coupling the Italian version of the RBD screening questionnaire (RBDSQ) using a cut-off of 8 [34] (patients were then allocated in PD + RBD or PD-RBD).

Within 2 weeks from enrollment, all participants underwent a structured psychiatric interview Structured Clinical Interview for DSM-5 Disorders—Clinician Version (SCID-5-CV), SCID-5 Research Version and SCID-5-Personality Disorders, for the identification of mental disorders [33]. All psychiatric diagnoses were made by a senior psychiatrist.

### 2.3. Neuropsychological and Neuropsychiatric Evaluation

All patients were submitted to a detailed neuropsychological evaluation [33], including: (1) the MMSE, a global index of cognitive impairment; (2) tests taken from the Mental Deterioration Battery, a comprehensive neuropsychological battery that includes verbal and non-verbal tasks such as the Rey’s 15-word test—Immediate Recall (RIR) and Delayed Recall (RDR) to evaluate short- and long-term episodic verbal memory, and the Phonological (PVF) and Semantic (SVF) Verbal Fluency tests to assess language abilities; (3) the Copy of the Rey–Osterrieth picture test (CRO) for evaluating complex constructional praxis; (4) the Wisconsin Card Sorting Test—Short Form (WCST-SF) to explore executive functions; and (5) the Stroop Word-Color Test (SWCT) to assess frontal abilities of simple attention, attention shifting and control.

The severities of symptoms of anxiety, alexithymia, apathy, anhedonia and depression were assessed in all participants [33]. Specifically, anxious symptomatology was quantified by the Hamilton Anxiety Rating Scale (HARS). Alexithymia was evaluated by the Toronto Alexithymia Scale-20 item (TAS-20). The TAS-20 comprises three subscales assessing different facets of alexithymia: F1, difficulty in identifying feelings; F2, difficulty in describing feelings; and F3, an externally oriented analytic mode of thinking. Apathy severity was quantified by means of the Apathy Rating Scale (ARS).

Hedonic tone was measured by the Snaith-Hamilton Pleasure Scale (SHAPS). Severity of depressive symptoms was investigated by the Beck Depression Inventory (BDI; total score) [35]. Cognitive performances and neuropsychiatric symptom severity were assessed by 3 trained neuropsychologists. Acceptable inter-rater reliability was defined as k > 0.80.

### 2.4. Statistical Analysis

Differences in sociodemographic and clinical characteristics among groups were assessed by Chi-square test and univariate analysis of variance (ANOVA) followed by Fisher’s Protected Least Significant Difference post-hoc tests, where appropriate. Post-hoc tests were repeated by Tukey’s Honestly Significant Difference test. Differences in clinical characteristics between PD + RBD and PD-RBD were analyzed using paired *t*-test. Levene’s test was used to test for equality variances.

To investigate differences in cognitive performances among diagnostic groups, we conducted a one-way multivariate analysis of variance (MANOVA) with a single independent variable (i.e., diagnosis) with four levels and 10 dependent variables (i.e., RIR, RDR, CRO, SWCT word reading, SWCT interference time, PVF, SVF, WCST-SF categories, WCST-SF perseverative errors, WCST-SF non-perseverative errors). The omnibus level of significance for MANOVA was set at *p* < 0.05. In the case of significant effect, we conducted a series of one-way ANOVAs followed by Fisher’s PLSD test post-hoc comparisons, when appropriate. The Bonferroni’s correction was applied before interpreting the significance of ANOVAs and of post-hoc tests, to control the risk of type I error. Post-hoc tests were repeated by Tukey’s HSD test. Cohen’s f was calculated to estimate effect size.

To investigate differences in severity of neuropsychiatric symptoms among groups, we conducted a MANOVA with a single independent variable (i.e., diagnosis) with four levels and five dependent variables (i.e., HARS, TAS-20, ARS, SHAPS and BDI Total score). The omnibus level of significance for MANOVA was set at *p* < 0.05. In the case of significant effects, we conducted a series of one-way ANOVAs followed by Fisher’s PLSD test post-hoc comparisons, when appropriate. The Bonferroni’s correction was applied before interpreting the significance of ANOVAs and of post-hoc tests, to control the risk of type I error. Post-hoc tests were repeated by Tukey’s HSD test. Cohen’s f was calculated to estimate effect size.

## 3. Results

### 3.1. Sociodemographic and Clinical Characteristics

As expected, we found a significantly higher score in the UPDRS-III in the two groups of patients diagnosed with PD compared to iRBD. No significant differences were found in illness duration, daily levodopa equivalent dosage and H&Y stage between the two groups diagnosed with PD (Table 1). 

### 3.2. Neuropsychological Assessment

MANOVA model indicated global difference (F_3.30_ = 8.337; *p* < 0.0001; and η^2^p = 0.246) in neuropsychological scores among groups.

**Figure 1:**
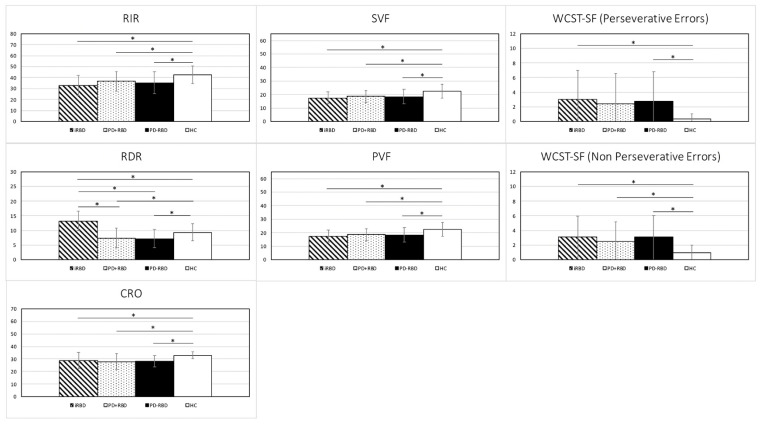
Significant neuropsychological differences among iRBD, PD + RBD, PD-RBD and HC groups. RBD = rapid eye movement sleep behavior disorder; iRBD = idiopathic RBD; PD = Parkinson’s disease; HC = healthy comparators; RIR = Rey’s 15-word test—Immediate Recall; RDR = Rey’s 15-word test—Delayed Recall; CRO = Copy of the Rey-Osterrieth picture test; PVF = Phonological Verbal Fluency; SVF = Semantic Verbal Fluency; and WCST-SF = Wisconsin Card Sorting Test –Short Form. * Significant difference (details are reported in Table 2).

As shown in Table 2 and Figure 1, iRBD patients scored worse than HC in RIR, CRO, PVF, SVF, WCST-SF perseverative and non-perseverative errors. No differences emerged between iRBD and the two PD groups. PD + RBD and PD-RBD performed worse than HC in all the evaluations, except for WCST-SF perseverative errors, where no differences were detected between PD + RBD and HC. No differences emerged between PD + RBD and PD-RBD. 

Surprisingly and intriguingly, iRBD showed a significantly higher score in RDR than PD + RBD, PD-RBD and HC.

### 3.3. Neuropsychiatric Assessment

As to neuropsychiatric characteristics, the MANOVA model showed global difference among groups (F_3.15_ = 3.053; *p* = 0.0001; and η^2^p = 0.740). 

**Figure 2:**
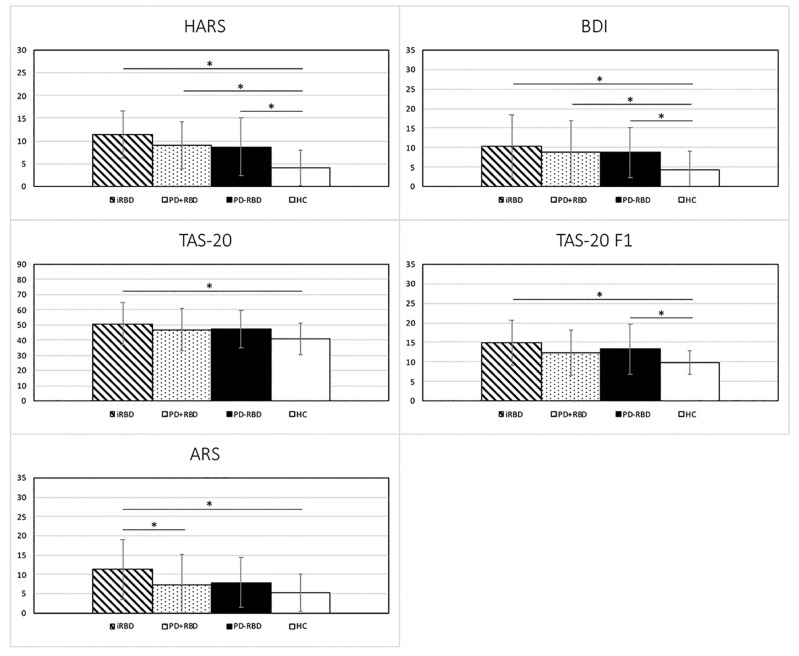
Significant neuropsychiatric differences among iRBD, PD + RBD, PD-RBD and HC groups. RBD = rapid eye movement sleep behavior disorder; iRBD = idiopathic RBD; PD = Parkinson’s disease; HC = healthy comparators; HARS = Hamilton Anxiety Rating Scale; TAS-20 = Toronto Alexithymia Scale—20 item; TAS-20 F1 = difficulty identifying feelings; ARS = Apathy Rating Scale; and BDI = Beck Depression Inventory. * Significant difference (details are reported in Table 3).

Patients with iRBD showed greater levels of anxiety, depression, apathy and alexithymia than HC. In particular, iRBD scored worse than HC in TAS-20 F1. Furthermore, iRBD did not differ from PD groups, except for higher levels of apathy than PD + RBD. Both PD groups showed grater levels of anxiety and depression than HC. PD-RBD had a worse score than HC in TAS-20 F1. No differences emerged between PD + RBD and PD-RBD (Table 3 and Figure 2).

## 4. Discussion

Increasing evidence indicates iRBD is a possible prodromal state of synucleinopathy. The occurrences of neurobiological abnormalities are considered precocious markers of phenoconversion. Thus, here we aimed to show specific cognitive and neuropsychiatric phenotypes in patients diagnosed with iRBD compared to PD and HC. 

Results of the present study confirm our hypothesis that patients with iRBD precociously experience cognitive and neuropsychiatric symptoms that are comparable to those subsequently observed in PD. Excluding that the possibility these symptoms are secondary to poor sleep quality [36], our findings claim prodromal neurodegeneration in iRBD. In particular: (1) iRBD and PD share poor global cognitive performance, with the exception of higher long term verbal memory score in iRBD; (2) iRBD has higher apathy in comparison to HC [15,16] and PD [18,20,21]; (3) iRBD and PD had more severe neuropsychiatric phenomenology than HC, namely depression and anxiety; and (4) iRBD has more severe alexithymia in comparison with HC.

Our results on poor performances in memory, language, praxis and executive functions of patients diagnosed with iRBD are confirmatory and consistent with the literature, showing a worse performance compared to HC and similar impairment compared to PD [37]. We also found a higher score in long-term verbal memory of iRBD, and this intriguing result must be subject of speculations. Indeed, it could be the result of an attempt for reorganization of reduced global cognitive reserve. Compensatory mechanisms prevent, or at least delay, the early drop in cognitive performance in iRBD, but not in more advanced neurodegeneration characterizing PD. In line with this hypothesis, Scherfler et al. [38] found increased grey matter volume, in iRBD compared to HC, in bilateral hippocampus and parahippocampal gyrus, areas implicated in the regulation of the REM sleep and involved in long-term memory. The increased volume may be the result of germination of new neural connections and/or strengthened pathways [39], both phenomena possibly determined by neuroplastic reorganization initiated by iRBD. Further, Mazza and colleagues [40], in a Single-photon Emission Computed Tomography study, described increased cerebral blood flow in the hippocampus of patients with iRBD. Thus, future neuroimaging studies are needed to confirm the above-mentioned hypotheses. Another interpretation could be taken into account. Several studies showed that patients with iRBD have more sleep slow-wave activity (SWA) than HC [41]. It is well established [42] that SWA is implicated in consolidation of hippocampus dependent episodic memory. Therefore, higher RDR score in iRBD could be a measurable neuropsychological correlate of augmented memories processes occurring in these patients. Here, we did not perform slow-wave sleep analysis, therefore future investigations on the relationships between sleep architecture changes and cognitive profile in patients with iRBD are needed.

Our results are in contrast with previous studies indicating long-term verbal memory deficit in iRBD [2,14,17,19]. However, these findings were obtained in patients with older age and long RBD duration, and the compensatory mechanisms we discussed here should be considered only in younger patients at the earlier phases of the illness. 

Our approach on the neuropsychiatric profile of patients with iRBD considers a comprehensive assessment and is noteworthy because it indicates the presence of a number of clinical symptoms. Specifically, our iRBD experience anxiety, depressive mood, alexithymia and apathy. This profile is in line with results described in previous studies [24,43,44] that, however, investigated individual neuropsychiatric symptoms separately. Thus, we clarify here for the first time in the same cohort, that iRDB has comprehensive neuropsychiatric phenomenology.

The occurrence of dream content abnormalities in RBD suggests patients may experience alexithymic symptoms related to missing imagery and lack introspection ability and propensity to adopt conformist behavior; all symptoms included in TAS-20 F3 independent dimension. In reality, previous evidence of alexithymia in iRBD [45,46] indicates difficulty in identifying feelings (TAS-20 F1) as the only alexithymic feature. We confirm this finding in iRBD and, as in our previous study [47], we report impairment in identifying feelings in patients with PD. Thus, our results on alexithymia in iRBD support, once again, the concept that iRBD shares many different characteristics with PD and may be considered as the prodromal phase of PD.

From a neurobiological perspective, alexithymic symptoms in iRBD could be linked to changes in the limbic circuit, with a greater involvement of prefrontal brain regions. These cerebral areas have been already described as possible mechanisms of iRBD [40]. Moreover, alexithymia in iRBD may be a psychological correlate of dysautonomia disorder [3,48]. Specifically, autonomic denervation would lead to reduction in heart rate variability and failure of the autonomic afferent pathways. Consequently, the decrease in incoming autonomic information may impair the ability to identify feelings associated with body sensation [45]. 

Our patients with iRBD experienced significantly more apathetic symptoms, even compared to PD. Apathy in iRBD is linked to the degeneration of dopaminergic neurons involved in motivation and reward/effort-based decision-making pathways [23]. In PD, there is strong evidence of dopamine dysfunction underlying apathy [49,50]; thus, a possible explanation of apathy results in our patients is that dopaminergic therapy in PD may have improved the apathetic symptomatology.

Overall, we found that our patients with iRBD are comparable with PD and more impaired than HC in almost all explored dimensions, indicating early onset of cognitive and neuropsychiatric dysfunctions during the prodromal phases of neurodegeneration. 

We acknowledge a number of issues that may limit the interpretations of some results of our study. A formal categorical diagnosis of Mild Cognitive Impairment (MCI) at the first visit was not performed here, because we used a continuous value approach on cognitive dimensions/symptoms. However, the presence of MCI is going to be considered as a possible early indicator of conversion from iRBD to a neurodegenerative disease [51,52]. Thus, further studies should clarify pros and cons of the two categorical/dimensional approaches. Additionally, v-PSG was not performed in our PD patients, but RBD diagnosis was made by a well-validated instrument (RBDSQ). Indeed, it is well described that the RBDSQ has high sensitivity and specificity to reliably screen RBD in PD [53]. Finally, our study is cross-sectional. To definitively investigate how important cognitive/neuropsychiatry symptoms in iRBD are as crucial factors for early diagnosis and conversion, longitudinal data are needed. However, the cross-sectional results may be also considered the main strength of our study, because the extensive neuropsychological and neuropsychiatric evaluation here applied demonstrated its value in the clinical manifestations of this prodromal neurodegenerative disorder. 

In conclusion, patients diagnosed with iRBD have specific cognitive and neuropsychiatric phenotypes characterized by poor global cognitive performance, but better long-term memory, and higher level of depression, anxiety, alexithymia and apathy. In particular, phenomenology of alexithymia and apathy in iRBD indicates precocious derangement of emotional regulation, subsequently observed also in PD. Although iRBD is a well-known predictive clinical manifestation of neurodegenerative disease onset, our results highlight that peculiar symptoms could be accepted as early clinical markers in development of PD or other types of neurodegenerative diseases, and should be routinely evaluated in clinical setting.

## Figures and Tables

**Table 1:** Sociodemographic and clinical characteristics of iRBD, PD + RBD, PD-RBD and HC groups.

Characteristics	iRBD	PD + RBD	PD-RBD	HC	*F*-value	df	*P*-value	Cohen’s f	post-hoc ***					
	(*n* = 38)	(*n* = 38)	(*n* = 38)	(*n* = 38)					iRBD vs. HC (Cohen’s f)	iRBD vs. PD + RBD (Cohen’s f)	iRBD vs. PD-RBD (Cohen’s f)	HC vs. PD + RBD (Cohen’s f)	HC vs. PD-RBD (Cohen’s f)	PD + RBD vs. PD-RBD (Cohen’s f)
Age ** (Years)	67.61 ± 6.99	67.16 ± 7.38	67.26 ± 7.18	67.47 ± 7.40	0.030	3	0.9931	n.a.	n.a.	n.a.	n.a.	n.a.	n.a.	n.a.
Education ** (Years)	11.87 ± 4.02	12.18 ± 3.99	11.29 ± 3.72	12.03 ± 4.00	0.0374	3	0.7719	n.a.	n.a.	n.a.	n.a.	n.a.	n.a.	n.a.
MMSE **	28.19 ± 1.67	28.58 ± 1.39	28.63 ± 1.48	29.32 ± 0.93	4.343	3	0.0058*	0.2919	0.0005 * (0.2867)	0.2178	0.1628	0.0223 * (0.1877)	0.0336 * (0.1750)	0.8691
Disease duration (Years)	~	4.68 ± 3.57	3.96 ± 3.12	~	0.917	1	0.3413	n.a.	n.a.	n.a.	n.a.	n.a.	n.a.	n.a.
Time from symptoms onset (Years)	5.08 ± 6.64	~	~	~	n.a.	n.a.	n.a.	n.a.	n.a.	n.a.	n.a.	n.a.	n.a.	n.a.
UPDRS-III score	2.37 ± 2.43	19.26 ± 10.30	16.66 ± 10.13	~	17.360	2	< 0.0001 *	0.8779	n.a.	< 0.0001 * (0.8152)	< 0.0001 * (0.6898)	n.a.	n.a.	0.2123
Hoehn &Yahr stage	~	1.97 ± 0.60	1.82 ± 0.60	~	1.314	1	0.2554	n.a.	n.a.	n.a.	n.a.	n.a.	n.a.	n.a.
Daily Levodopa Equivalent Dose (mg)	~	486.18 ± 370,66	388.12 ± 363.01	~	1.358	1	0.2477	n.a.	n.a.	n.a.	n.a.	n.a.	n.a.	n.a.
Characteristic	iRBD	PD + RBD	PD-RBD	HC	Chi	df	*P*-value	Cohen’s f	post-hoc					
	(*n* = 38)	(*n* = 38)	(*n* = 38)	(*n* = 38)					iRBD vs. HC	iRBD vs. PD + RBD	iRBD vs. PD-RBD	HC vs. PD + RBD	HC vs. PD-RBD	PD + RBD vs. PD-RBD
Sex (n. Male/n. Female)	31/7	31/7	31/7	31/7	0	3	n.a.	n.a.	n.a.	n.a.	n.a.	n.a.	n.a.	n.a.

Data represent mean ± SD (Mean Rank ± Standard Deviation); RBD = rapid eye movement sleep behavior disorder; iRBD = idiopathic RBD; PD = Parkinson’s disease; HC = healthy comparators; MMSE = Mini-Mental State Examination; and UPDRS-III = Unified Parkinson’s Disease Rating Scale—Part III; * Significant at *p* < 0.05; ** Results of Levene’s test (Levene Statistic; df1; df2; and *p*-value) for age (0.096; 3; 148; and 0.962), education (0.045; 3; 148; and 0.987) and MMSE (2.888; 3; 148; and 0.038); *** Tukey’s HSD analysis replicates exactly the results of Fisher’s LSD test Bonferroni corrected shown here. Statistical parameters available upon request.

**Table 2:** Neuropsychological characteristics of iRBD, PD + RBD, PD-RBD and HC groups.

Variables	iRBD	PD + RBD	PD-RBD	HC	*F*-value	df	*P*-value	Cohen’s f	post-hoc ***					
	(*n* = 38)	(*n* = 38)	(*n* = 38)	(*n* = 38)					iRBD vs. HC (Cohen’s f)	iRBD vs. PD + RBD (Cohen’s f)	iRBD vs. PD-RBD (Cohen’s f)	HC vs. PD + RBD (Cohen’s f)	HC vs. PD-RBD (Cohen’s f)	PD + RBD vs. PD-RBD (Cohen’s f)
RIR	32.74 ± 9.00	36.55 ± 8.57	35.37 ± 10.14	42.66 ± 8.14	8.291	3	< 0.0001 *	0.4045	< 0.0001 ** (0.3900)	0.0664 (0.1498)	0.2041 (0.1034)	0.0036 ** (0.2402)	0.0005 ** (0.2866)	0.5668 (0.0464)
RDR	13.21 ± 3.35	7.37 ± 2.79	7.24 ± 3.03	9.34 ± 2.88	32.315	3	< 0.0001 *	0.7986	< 0.0001 ** (0.4531)	< 0.0001 ** (0.6838)	< 0.0001 ** (0.6990)	0.0050 ** (0.2307)	0.0028 ** (0.2459)	0.8497 (0.0152)
CRO	28.64 ± 6.39	27.82 ± 5.12	28.32 ± 4.61	32.88 ± 2.72	8.675	3	< 0.0001 *	0.4134	0.0002 ** (0.3065)	0.4611 (0.0593)	0.7698 (0.0231)	< 0.0001 ** (0.3658)	< 0.0001 ** (0.3296)	0.6565 (0.0361)
SWCT (Word Reading)	15.92 ± 3.61	15.58 ± 4.40	15.74 ± 3.77	14.05 ± 3.09	1.993	3	0.1176	n.a.	n.a.	n.a.	n.a.	n.a.	n.a.	n.a.
SWCT (Color Naming)	21.82 ± 5.18	22.10 ± 5.87	21.63 ± 5.66	19.37 ± 3.79	2.23	3	0.0875	n.a.	n.a.	n.a.	n.a.	n.a.	n.a.	n.a.
SWCT (Interference)	50.63 ± 26.23	49.79 ± 30.14	43.34 ± 12.48	38.24 ± 10.69	2.773	3	0.0436	n.a.	n.a.	n.a.	n.a.	n.a.	n.a.	n.a.
PVF	30.90 ± 11.23	31.55 ± 9.11	28.13 ± 7.44	38.97 ± 11.40	8.273	3	<0.0001 *	0.4040	0.0005 ** (0.2873)	0.7732	0.2271	0.0014 ** (0.2642)	< 0.0001 ** (0.3860)	0.1353
SVF	17.53 ± 4.48	18.66 ± 5.03	18.37 ± 5.27	22.40 ± 5.00	7.228	3	0.0001 *	0.3777	< 0.0001 ** (0.3476)	0.3208	0.4596	0.0013** (0.2670)	0.0005 ** (0.2877)	0.7992
WCST-SF (Categories)	5.42 ± 1.18	5.58 ± 1.00	5.37 ± 1.10	6.00 ± 0.00	3.456	3	0.0181	n.a.	0.0087	0.4696	0.8094	0.0551	0.0043	0.3353
WCST-SF (Perseverative Errors)	3.03 ± 3.98	2.42 ± 4.14	2.79 ± 4.07	0.37 ± 0.71	4.480	3	0.0048 *	0.2974	0.0013** (0.2659)	0.4569	0.7708	0.0125	0.0033 ** (0.2419)	0.6505
WCST-SF (Non perseverative Errors)	3.10 ± 2.86	2.55 ± 2.64	3.08 ± 2.96	0.95 ± 1.01	6.299	3	0.0005 *	0.3513	0.0002** (0.3045)	0.3354	0.9634	0.0057 ** (0.2266)	0.0003 ** (0.3017)	0.3554

Data represent mean ± SD (Mean Rank ± Standard Deviation); RBD = rapid eye movement sleep behavior disorder; iRBD = idiopathic RBD; PD = Parkinson’s disease; HC = healthy comparators; RIR = Rey’s 15-word test—Immediate Recall; RDR = Rey’s 15-word test—Delayed Recall; CRO = Copy of the Rey–Osterrieth picture test; SWCT = Stroop Word-Color Test; PVF = Phonological Verbal Fluency; SVF = Semantic Verbal Fluency; WCST-SF = Wisconsin Card Sorting Test –Short Form; * Significant at *p* < 0.005; ** Significant at *p* < 0.008; and *** Tukey’s HSD analysis replicates exactly the results of Fisher’s LSD test Bonferroni corrected shown here. Statistical parameters available upon request.

**Table 3:** Neuropsychiatric characteristics of iRBD, PD + RBD, PD-RBD and HC groups.

Variables	iRBD	PD + RBD	PD-RBD	HC	*F*-value	df	*P*-value	Cohen’s f	post-hoc ***					
	(*n* = 38)	(*n* = 38)	(*n* = 38)	(*n* = 38)					iRBD vs. HC (Cohen’s f)	iRBD vs. PD + RBD (Cohen’s f)	iRBD vs. PD-RBD (Cohen’s f)	HC vs. PD + RBD (Cohen’s f)	HC vs. PD-RBD (Cohen’s f)	PD + RBD vs. PD-RBD (Cohen’s f)
HARS	11.42 ± 5.12	9.05 ± 5.07	8.71 ± 6.41	4.10 ± 3.94	13.114	3	< 0.0001 *	0.5089	< 0.0001 ** (0.4968)	0.0493	0.0247	< 0.0001 ** (0.3360)	0.0002 ** (0.3129)	0.7750
TAS-20	50.55 ± 13.84	46.87 ± 11.27	47.34 ± 12.07	40.76 ± 10.17	4.474	3	0.0049 *	0.2972	0.0005 ** (0.2906)	0.1797	0.2420	0.0270	0.0173	0.8626
TAS-20 F1	14.97 ± 5.79	12.32 ± 5.16	13.29 ± 6.37	9.79 ± 3.06	6.491	3	0.0004 *	0.3575	< 0.0001 ** (0.3491)	0.0287	0.1636	0.0374	0.004 ** (0.2359)	0.4196
TAS-20 F2	14.10 ± 6.11	13.71 ± 5.70	14.05 ± 5.93	11.76 ± 5.14	1.426	3	0.2377	n.a.	n.a.	n.a.	n.a.	n.a.	n.a.	n.a.
TAS-20 F3	21.47 ± 5.97	20.84 ± 5.01	20 ± 5.91	19.21 ± 5.19	1.207	3	0.3092	n.a.	n.a.	n.a.	n.a.	n.a.	n.a.	n.a.
ARS	11.24 ± 7.77	7.32 ± 5.55	7.90 ± 6.42	5.24 ± 4.73	6.081	3	0.0006 *	0.3464	< 0.0001 ** (0.3411)	0.0067 ** (0.2228)	0.0205	0.1472	0.0644	0.6855
SHAPS	0.32 ± 0.78	0.29 ± 0.57	0.40 ± 0.68	0.24 ± 0.43	0.420	3	0.7390	n.a.	n.a.	n.a.	n.a.	n.a.	n.a.	n.a.
BDI Total score	10.37 ± 7.99	8.92 ± 5.76	8.82 ± 6.40	4.32 ± 4.69	6.543	3	0.0003 *	0.3591	< 0.0001 ** (0.3382)	0.3200	0.2862	0.0018 ** (0.2572)	0.0023 ** (0.2516)	0.9423

Data represent mean ± SD (Mean Rank ± Standard Deviation); iRBD = idiophatic rapid eye movement REM sleep behavior disorder; iRBD = idiopathic RBD; PD = Parkinson’s disease; HC = healthy comparators; HARS = Hamilton Anxiety Rating Scale; TAS-20 = Toronto Alexithymia Scale—20 item; TAS-20 F1 = difficulty identifying feelings; TAS-20 F2 = difficulty describing feelings; TAS-20 F3 = externally oriented thinking; ARS = Apathy Rating Scale; SHAPS = Snaith Hamilton Pleasure Scale; BDI = Beck Depression Inventory; * Significant at *p* < 0.01; ** Significant at *p* < 0.008; and *** Tukey’s HSD analysis replicates exactly the results of Fisher’s LSD test Bonferroni corrected shown here. Statistical parameters available upon request.

## Data Availability

The data presented in this study are available on request from the corresponding author. The data are not publicly available due to internal policy.
